# A Longitudinal Cohort to Monitor Malaria Infection Incidence during Mass Drug Administration in Southern Province, Zambia

**DOI:** 10.4269/ajtmh.19-0657

**Published:** 2020-07-02

**Authors:** Adam Bennett, Travis R. Porter, Mulenga C. Mwenda, Joshua O. Yukich, Timothy P. Finn, Chris Lungu, Kafula Silumbe, Brenda Mambwe, Sandra Chishimba, Conceptor Mulube, Daniel J. Bridges, Busiku Hamainza, Laurence Slutsker, Richard W. Steketee, John M. Miller, Thomas P. Eisele

**Affiliations:** 1 Malaria Elimination Initiative, Global Health Group, University of California San Francisco, San Francisco, California;; 2 Department of Tropical Medicine, Center for Applied Malaria Research and Evaluation, Tulane University School of Public Health and Tropical Medicine, New Orleans, Louisiana;; 3 PATH Malaria Control and Elimination Partnership in Africa (MACEPA), Lusaka, Zambia;; 4 National Malaria Elimination Centre, Zambia Ministry of Health, Lusaka, Zambia;; 5 PATH MACEPA, Seattle, Washington

## Abstract

Rigorous evidence of effectiveness is needed to determine where and when to apply mass drug administration (MDA) or focal MDA (fMDA) as part of a malaria elimination strategy. The Zambia National Malaria Elimination Centre recently completed a community-randomized controlled trial in Southern Province to evaluate MDA and fMDA for transmission reduction. To assess the role of MDA and fMDA on infection incidence, we enrolled a longitudinal cohort for an 18-month period of data collection including monthly malaria parasite infection detection based on polymerase chain reaction and compared time to first infection and cumulative infection incidence outcomes across study arms using Cox proportional hazards and negative binomial models. A total of 2,026 individuals from 733 households were enrolled and completed sufficient follow-up for inclusion in analysis. Infection incidence declined dramatically across all study arms during the period of study, and MDA was associated with reduced risk of first infection (hazards ratio: 0.36; 95% CI: 0.16–0.80) and cumulative infection incidence during the first rainy season (first 5 months of follow-up) (incidence rate ratio: 0.34; 95% CI: 0.12–0.95). No significant effect was found for fMDA or for either arm over the full study period. Polymerase chain reaction infection status at baseline was strongly associated with follow-up infection. The short-term effects of MDA suggest it may be an impactful accelerator of transmission reduction in areas with high coverage of case management and vector control and should be considered as part of a malaria elimination strategy.

## INTRODUCTION

Over the past decade, Zambia’s National Malaria Elimination Centre (NMEC) has scaled up the national coverage of proven vector control and case management interventions, and expanded a network of community health workers for community case management, rapid reporting, and active surveillance.^1^ As a result of these successes, the NMEC has progressed to an elimination strategy in Southern Province and has recently conducted a community-randomized controlled trial to assess the impact of four rounds of mass drug administration (MDA) or focal MDA (fMDA) using dihydroartemisinin–piperaquine (DHAp) compared with standard of care interventions.^2^ In this trial, MDA consisted of administration of DHAp to all eligible household members in selected health facility catchment areas (HFCAs), whereas fMDA in selected HFCAs limited administration of DHAp to households where at least one individual tested positive by malaria rapid diagnostic test (RDT).

As part of this trial, a longitudinal cohort was enrolled to measure the impact of MDA or fMDA on infection incidence over an 18-month period. Given their expense and complex data collection requirements, longitudinal cohort studies are performed infrequently yet provide a valuable source of information on intervention efficacy and force of infection over time and within specific subpopulations. Although several studies have examined clinical incidence or parasite prevalence measures, only one previous randomized controlled trial of MDA has included actively detected community-level measures of infection incidence over an extended follow-up period,^3^ and few previous studies have included long-term active follow-up of infection incidence over multiple rounds of MDA.^4,5^ Clinical incidence recorded through the health information system provides an important data point that may not only reflect force of infection^6^ but is also subject to biases including variability in human immunity and clinical response, treatment seeking, and quality of reporting. Similarly, periodic estimates of parasite prevalence are valuable for estimating long-term trends in transmission,^7^ but cross-sectional “snapshots” may be influenced by short-term and heterogeneous fluctuations in factors that influence transmission, such as rainfall and access to treatment. The longitudinal data presented in this article fill an important gap and provide additional context to results presented elsewhere.^8^

Mass drug administration has been recommended for use in specific settings including during epidemics and for areas approaching elimination,^9^ but limited rigorous evidence exists on the impact of differing MDA strategies across a broad range of epidemiological settings. In this study, we sought to examine the effect of MDA or fMDA with DHAp on the time to first infection and cumulative infection incidence, stratified by high and low transmission and in specific time periods determined by malaria seasonality. In addition, we sought to evaluate environment-, household-, and individual-level risk factors associated with infection incidence.

## METHODS

### Study site.

The full protocol for the trial, as well as detailed methods and a description of the study site along Lake Kariba in Southern Province, Zambia, is described elsewhere and in this supplement.^2,10^ The cohort study was conducted in all 60 HFCAs included in the trial, of which 20 each were randomized to MDA, fMDA, or control. The trial area was equally stratified a priori into higher (> 10% parasite prevalence in children younger than 5 years) and lower (< 10% parasite prevalence in children younger than 5 years) transmission strata at randomization. Malaria transmission in the study site is seasonal, with the high transmission season corresponding with seasonal rains occurring between December and May.

### Sample selection.

A complete household enumeration with global positioning system (GPS) units was conducted in 2013 and 2014 to create the study sample frame, which included a listing of usual household members for each household within the trial HFCAs, totaling roughly 56,000 households and 330,000 individuals. To facilitate field data collection logistics, a 5-km^2^ buffer was created around each health facility point, and households were considered eligible for selection into the cohort if they fell within this buffer and had at least three household members. A total of 13 households were then randomly selected within each HFCA. Within each selected household, two individuals older than 3 months of age but younger than 20 years, and one older than 20 years, were randomly selected from the household listing for inclusion. A listing of alternate individuals was provided for each household. In some catchment areas, there were not 13 households with eligible individuals in each age category within 5 km^2^ of the health facility. In these cases, the area was expanded until at least 13 households were available.

### Data collection.

Data collection was conducted between December 2014 and May 2016 (a total of 18 months). Cohort participants were enrolled in December 2014 coinciding with the first MDA/fMDA intervention round. During each month of data collection, community health workers visited selected households, conducted a brief questionnaire with consenting cohort enrollees, and collected blood samples for testing via RDT and two dried blood spots for polymerase chain reaction (PCR) analysis. In addition to December 2014, other cohort months coincided with MDA/fMDA intervention rounds (February 2015, October 2015, and February 2016); during these months, all members of the household were given their randomly assigned treatment group exposure, meaning individuals in the MDA and fMDA HFCAs received these interventions as per the trial protocol. In control areas and all other months, any individuals in the cohort testing positive by RDT were administered artemether-lumefantrine (AL) as per the national treatment policy.

The questionnaire collected information on household assets, household indoor residual spraying (IRS) in the previous 12 months, long-lasting insecticide-treated net (LLIN) coverage, any recent fever, treatment-seeking and medication received for fever, and any recent travel. Data were recorded in personal digital assistants, with individual visit and RDT results also recorded on paper forms. GPS points of all households were collected at baseline, and altitude (in meters), monthly rainfall (mm), and environmental vegetation index were included for each household from remote-sensing data sources.^11^ A household wealth index was created from the household asset listing using principal components analysis and Euclidean distance to the nearest health facility, and permanent water body was calculated for each household.

### Primary outcomes.

The primary outcomes for the analysis included time to first infection, defined as the number of months between baseline and the first PCR-positive test per individual, and cumulative infection incidence, defined as the total number of monthly PCR-positive tests per individual divided by the total number of tests conducted per individual per time period.

### Data analysis.

Cohort months were numbered and defined as baseline (December 2014) or follow-up (January 2015–May 2016). Individuals were considered enrolled in the cohort if they had a first month RDT or PCR value, but were only included in follow-up data analyses if they had data collected during at least three of the first 6 months of follow-up (January–June 2015), so as to ensure consistent data over the initial period of follow-up; all other individuals were dropped from the analysis. In addition, individuals were removed from the follow-up analysis if they were later determined to have had an infection by PCR at baseline but were RDT negative or RDT missing at baseline and did not report receiving treatment, as these individuals would not have had their infections cleared. During the follow-up period, positive PCR values were removed if they occurred following a positive in the previous month, and during the previous month no treatment was reported. Descriptive analysis of infection incidence rates was conducted by first summing total positive PCR results per time period of follow-up and dividing by the total number of months with data per individual. Descriptive statistics were examined, for each study arm, by age category, gender, high/low transmission, and time period of follow-up reflecting rainy (January–May 2015, December 2015–May 2016) and dry seasons (June–November 2015). Climatic, geographic, and intervention coverage variables were examined for differences between study arms at baseline.

All analyses were performed as intention-to-treat, whereby all individuals allocated to MDA, fMDA, or control arms were included in these arms for analysis, irrespective of individual treatment. Comparison of time-to-event outcomes between intervention arms was conducted using Kaplan–Meier curves and with unadjusted and adjusted Cox proportional hazards regression. Because of dramatic regional declines in transmission during the trial period, as well as marked seasonality, we examined separate time-to-event models for the first rainy season of the study period as well as for the full study period. In these analyses, all individuals entered the analysis during the first month of inclusion after baseline and exited when they either experienced an event (positive PCR test) or were censored at the end of data collection (end of May 2015 for the first rainy season or end of May 2016 for the full time period). The Cox proportional hazards models included a shared frailty at the health catchment level to account for unobserved cluster-level heterogeneity, and the adjusted model included covariates for the following potential risk factors: PCR infection status at baseline, age, gender, wealth quintile, household IRS at baseline, elevation (standardized to 1 SD), and the previous month’s rainfall and environmental vegetation index (standardized to 1 SD). Covariates including distance to the nearest health facility, and distance to Lake Kariba were also evaluated for inclusion. Hazard ratios (HRs) were used to compare time-to-event by study arm and evaluate covariate effects. Separate models were evaluated for high and low transmission strata, as well as for both strata combined. In addition, to assess the influence of the time from intervention on effect estimates, we examined models with increasing months censored, starting from 1 month after the second intervention round.

A comparison of cumulative infection incidence between intervention arms was conducted using unadjusted and adjusted negative binomial regression models, where the outcome was the count of infections identified per time period. These models included random effects at both the individual and health catchment levels to account for clustering and differential observation time. To examine specific effects of the study intervention by season, the primary unadjusted model included the study arm, season (rainy/dry), and an interaction term between the study arm and season (rainy/dry); a second unadjusted model with only the study arm was used to assess the entire period of study. Corresponding adjusted models included covariates for the following potential risk factors: PCR infection status at baseline, age, gender, wealth quintile, household IRS at baseline, elevation (standardized), mean rainfall (standardized) over the period of study, and mean environmental vegetation index (standardized) over the period of study. Covariates including distance to the nearest health facility and distance to Lake Kariba were also evaluated for inclusion. Incidence rate ratios (IRRs) were used to compare infection incidence by study arm and evaluate covariate effects; linear combinations were used to calculate time period–specific effects from interaction terms. Separate negative binomial regression models were conducted for high and low transmission strata, as well as both strata combined.

## RESULTS

### Baseline characteristics and descriptive statistics.

**Figure 1.**
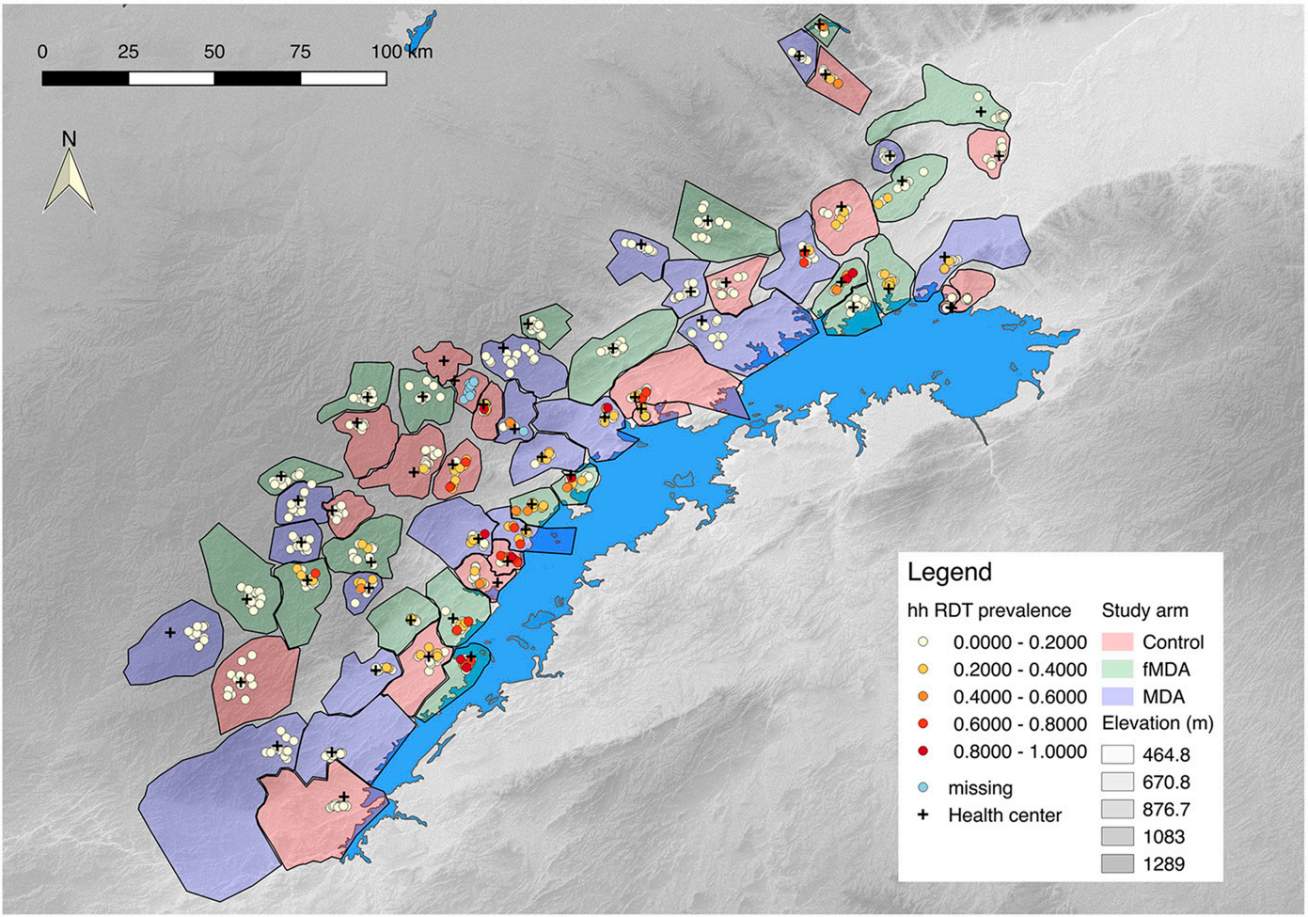
Map of study site including study health facility catchments by arm and baseline rapid diagnostic test (RDT) prevalence in cohort households. Baseline data for one control catchment were entirely missing, and baseline RDT values for the neighboring control catchment were missing (indicated in legend).

A total of 2,230 individuals from 742 households were successfully enrolled into the cohort study at baseline, and there were no systematic differences in individual, household, or environmental characteristics across study arms (Table 1). The proportion of individuals in homes with IRS in the previous 12 months at baseline was slightly higher in the fMDA and control arms, and the proportion of individuals in homes with at least one LLIN was highest in the MDA arm and lowest in the fMDA arm. The proportion of individuals above the median wealth score was highest in the fMDA arm and lowest in the MDA arm. The mean distance to the nearest facility, mean distance to the nearest water body, and mean altitude were similar across arms. Rapid diagnostic test positivity of cohort participants at baseline was higher in both the fMDA (9.3%) and control (8.2%) arms than the MDA arm (5.7%). Similarly, PCR positivity was highest in the fMDA arm (10.9%) and lower in the control (6.7%) and MDA arms (5.1%). Households with higher RDT positivity at baseline were clustered along the shore of Lake Kariba (Figure 1).

**Table 1:** Baseline characteristics of enrolled cohort population by study arm, Southern Province, Zambia, December 2014

Characteristic	Control	Mass drug administration	Focal mass drug administration
Age (years), *n* (%)			
< 5	134 (19.3)	157 (20.1)	141 (18.7)
5–19	311 (44.8)	355 (45.5)	352 (46.6)
> 19	249 (35.9)	269 (34.4)	262 (34.7)
Gender, *n* (%)			
Male	316 (45.5)	396 (50.7)	360 (47.7)
Female	378 (54.5)	385 (49.3)	395 (52.3)
Transmission stratum, *n* (%)			
High	368 (53.0)	371 (47.5)	363 (48.1)
Low	326 (47.0)	410 (52.5)	392 (51.9)
% In HH with indoor residual spraying at baseline	7.3 (3.3–15.6)	4.6 (2.1–9.9)	11.5 (4.5–26.4)
% In HH with ITN at baseline	72.3 (58.1–83.1)	82.3 (68.9–90.7)	62.1 (45.4–76.4)
% Above median SES	52.8 (43.5–62.0)	46.1 (38.9–53.5)	55.1 (47.0–62.8)
% Reporting travel in previous 2 weeks	4.3 (2.7–6.7)	6.7 (3.0–14.5)	6.5 (4.3–9.6)
Mean distance to health facility (km)	3.2 (2.6–3.9)	2.8 (1.9–3.7)	2.9 (2.3–3.4)
Mean distance to water body (km)	21.1 (13.2–29.1)	24.7 (16.4–32.9)	25.0 (15.2–34.7)
Mean altitude (m)	802.4 (646.7–958.0)	864.9 (721.4–1008.5)	851.7 (686.6–1016.8)
Mean environmental vegetation index (EVI)	0.19 (0.17–0.20)	0.18 (0.17–0.19)	0.18 (0.17–0.19)
Baseline % rapid diagnostic test+	8.5 (4.2–16.4)	5.7 (3.1–10.5)	9.3 (5.3–16.1)
Baseline % polymerase chain reaction	6.7 (3.4–12.8)	5.1 (2.6–9.8)	10.9 (6.1–18.7)
Total, *N*	694	781	755

HH = household; ITN = insecticide-treated net; SES = socio-economic status.

**Figure 2.**
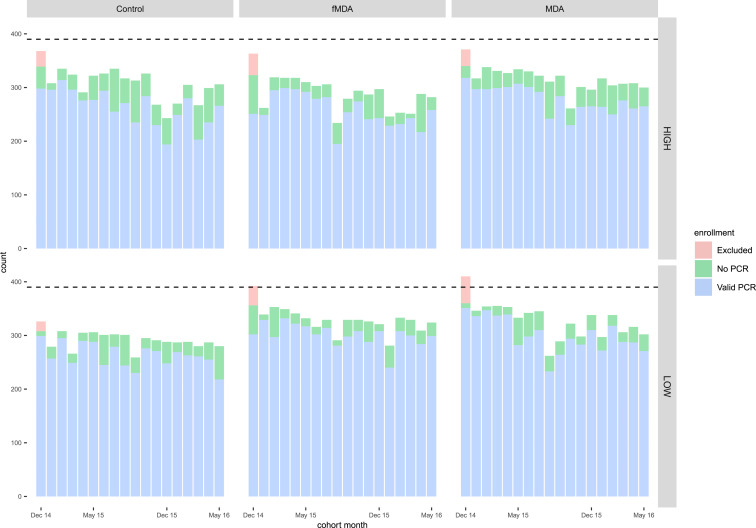
Among individuals enrolled at baseline, total number of individuals with data by month over the 18 months of data collection, by transmission strata and study arm. Bar plots indicate numbers of individuals with data for each month, and among those with adequate follow-up, bar plots indicate numbers with or without a PCR value by month. Dotted line indicates target enrollment per month.

A total of 2,054 (92.1%) of the enrolled individuals from 733 households completed at least three of the first 6 months of follow-up. One control catchment was removed from follow-up analyses because of missing baseline data, and 28 individuals with a PCR infection at baseline but either a negative or missing RDT result and no treatment data were removed. As a result, a total of 2,026 individuals from 733 households were included in follow-up analyses. Among these individuals, the mean monthly follow-up was similar across study arms, and 14.9 months overall; 97.3%, 89.3%, and 38.6% of individuals completed at least 6, 12, and 17 months of follow-up, respectively. There were no statistically significant (*P* < 0.05) differences in baseline characteristics between individuals who completed at least 3 of the first 6 months of follow-up and those who did not. Among all individuals included in follow-up analyses, the number of individuals followed up by month did not vary dramatically by arm or transmission strata, and valid PCR results were available for 90.0% of person-months (Figure 2).

### Infection incidence over follow-up period.

**Figure 3.**
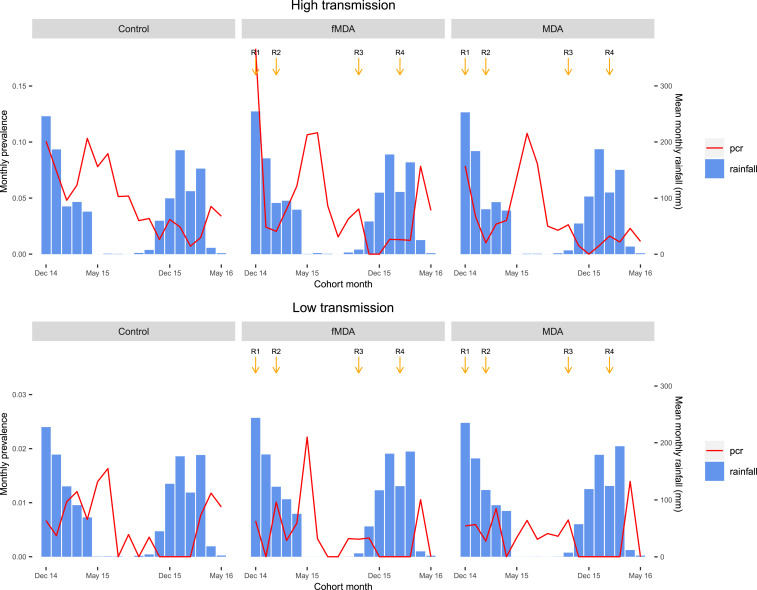
Monthly infection rates for polymerase chain reaction (PCR) and mean rainfall over the period of study, by transmission strata and study arm. Timing of each intervention round is indicated by downward arrows. This figure appears in color at www.ajtmh.org.

**Figure 4.**
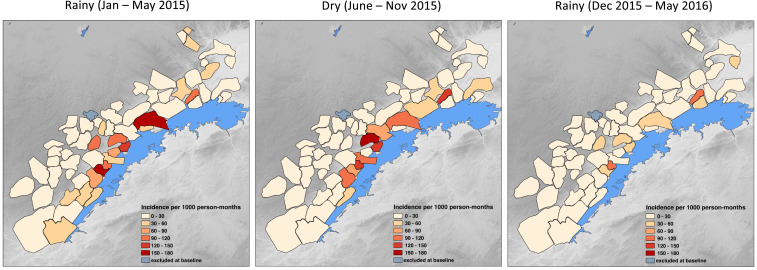
Polymerase chain reaction–based infection incidence rates per health facility catchment by season over the follow-up period, Southern Province, Zambia, January 2015–May 2016. Baseline month (December 2014) removed from first panel.

Overall, infection incidence measured by PCR decreased across all study arms during the 18 months of the cohort study. During the first rainy season follow-up period, cumulative PCR infection incidence was highest in the control arm (41.5 positives per 1,000 person-months) and lowest in the MDA arm (18.5 positives per 1,000 person-months) (Table 2). During the dry season, infection incidence was similar in the control (25.1 positives per 1,000 person-months) and MDA arms (25.0 positives per 1,000 person-months). Polymerase chain reaction infection incidence was lowest in the last rainy season period in the MDA arm (6.6 positives per 1,000 person-months) and highest in the control arm (14.6 positives per 1,000 person-months). Seasonality trends for monthly PCR positivity rates were similar across study arms and transmission levels (Figure 3), but there was substantial heterogeneity across study catchments within strata and arms. At the catchment level, higher incidence was clustered in catchments along the shore of Lake Kariba throughout the study, and the number of catchments with no infections increased from 18 during the first follow-up rainy season to 27 during the dry season and 34 during the final rainy season (Figure 4).

**Table 2:** Total follow-up infections per person-time for cohort population from polymerase chain reaction samples collected by individual characteristic, stratum, time period, and study arm; values indicate infections/1,000 person-time (number of infections/person-time), Southern Province, Zambia, January 2015–May 2016

Characteristic	Control	Mass drug administration	Focal mass drug administration
Age (years)			
< 5	19.7 (35/1,774)	19.3 (40/2,072)	22.6 (42/1,856)
5–19	33.7 (130/3,862)	20.2 (88/365)	16.5 (70/4,254)
> 19	22.6 (73/3,227)	13.2 (44/3,342)	24.0 (81/3,381)
Gender			
Male	31.6 (127/4,022)	15.5 (78/5,044)	16.4 (73/4,464)
Female	22.9 (111/4,841)	17.9 (84/4,691)	23.9 (120/5,027)
Transmission stratum			
High	47.9 (212/4,426)	30.8 (144/4,668)	39.6 (173/4,366)
Low	5.9 (26/4,437)	3.6 (18/5,067)	3.9 (20/5,125)
Season			
Rainy (January 2015–May 2015)	41.5 (117/2,819)	18.5 (58/3,140)	28.4 (86/3,026)
Dry (June 2015–November 2015)	25.1 (78/3,105)	25.0 (82/3,278)	20.6 (68/3,308)
Rainy (December 2015–May 2016)	14.6 (43/2,939)	6.6 (22/3,317)	12.4 (39/3,157)
Total follow-up	26.9 (238/8,863)	16.6 (162/9,735)	20.3 (193/9,491)

The number of infections per individual over the follow-up period varied by transmission strata, study arm, and baseline PCR status, with the number of infections per individual ranging from 0 to 7. Among individuals with a valid PCR result at baseline, 16.0% had at least one infection during follow-up, and 5.5% had more than one infection. Individuals who were PCR positive at baseline were far more likely to have at least one follow-up infection (46.7%) than those who were PCR negative at baseline (14.1%). For individuals who were PCR negative at baseline, there were similar proportions of individuals with ≥ 1 follow-up infections across study arms (Table 3). Conversely, among individuals who were PCR positive at baseline, 24.0% in the MDA arm had ≥ 1 follow-up infections, as compared with 66.7% and 47.8% in the control and fMDA arms, respectively.

**Table 3:** Proportion of individuals with at least one PCR follow-up infection, by baseline PCR status, transmission stratum, and arm for cohort population, Southern Province, Zambia, December 2014–May 2016

Proportion with any follow-up infection	High transmission				Low transmission			
	Control	MDA	fMDA	All	Control	MDA	fMDA	All
Baseline PCR (−)								
% Any infection (*n*)	25.0 (67)	25.5 (74)	25.9 (53)	25.4 (194)	5.8 (17)	4.0 (14)	5.0 (15)	4.9 (46)
Total, *N*	268	290	205	763	293	348	300	941
Baseline PCR (+)								
% Any infection (*n*)	66.7 (20)	24.0 (6)	47.8 (22)	47.5 (48)	50.0 (1)	0.0 (0)	50.0 (1)	33.3 (2)
Total, *N*	30	25	46	101	2	2	2	6

fMDA = focal mass drug administration; PCR = polymerase chain reaction; MDA = mass drug administration.

Follow-up infections were clustered within households, with only 29.3% of households having at least one infected individual, and 13.0% of households with more than one infected individual. Households with at least one infection at baseline were far more likely to have at least one infection during follow-up (64.6%) than households without an infection at baseline (22.5%). An individual’s risk of infection over the follow-up period was influenced by having any other infections in the household at baseline: among individuals who were PCR negative at baseline, an infection at baseline among any other member of the household was associated with having ≥ 1 follow-up infection (Table 4); this association was much stronger in the lower transmission strata (OR: 4.24; 95% CI: 1.05–17.1) than in the higher transmission strata (odds ratio [OR]: 1.70; 95% CI: 1.07–2.70).

**Table 4:** Proportion of individuals with at least one PCR follow-up infection, among those with no infection (PCR−) at baseline, stratified by households with or without another PCR + individual at baseline and transmission stratum, Southern Province, Zambia, December 2014–January 2016

Follow-up PCR status, among individuals PCR (−) at baseline	High transmission		Low transmission	
	No baseline HH PCR+	≥ 1 Baseline HH PCR+	No baseline HH PCR+	≥ 1 baseline HH PCR+
% No PCR+ (*n*)	78.0 (482)	60.0 (87)	95.5 (884)	73.3 (11)
% Any PCR + follow-up (*n*)	22.0 (136)	40.0 (58)	4.5 (42)	26.7 (4)
Total, *N*	618	145	926	15

### Time to first infection models.

**Figure 5.**
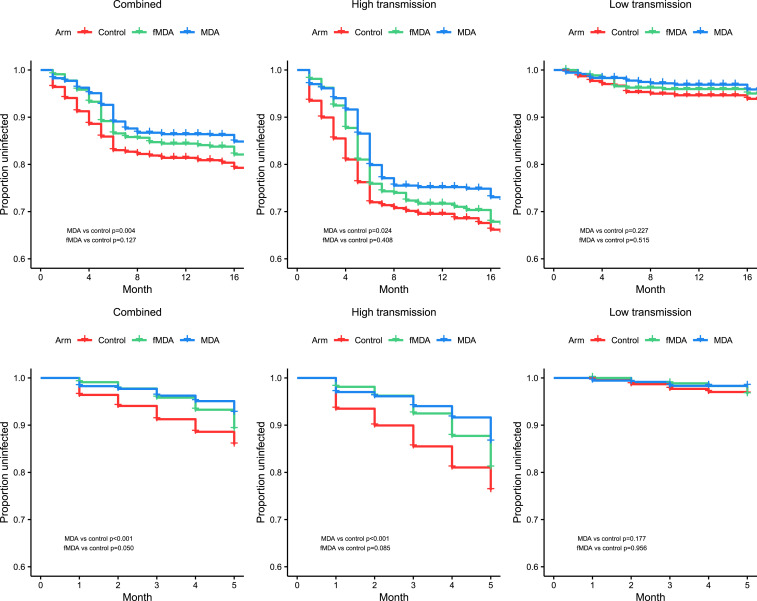
Kaplan–Meier curves by study arm for all catchments combined and high and low transmission strata over the full 17-month cohort study follow-up period (January 2015–May 2016) (top panel) and over the first rainy season only (January 2015–May 2015) (bottom panel). *P*-values represent results of log-rank test for each arm vs. control.

**Figure 6.**
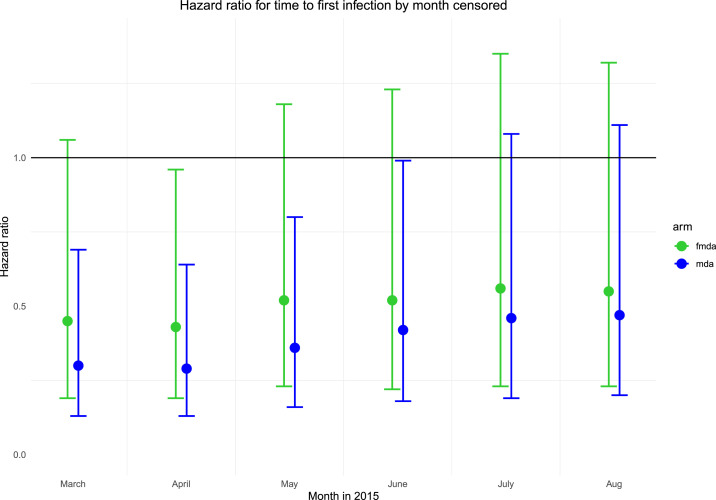
Hazard ratio for mass drug administration (MDA) and focal mass drug administration (fMDA) compared with control from adjusted Cox proportional hazards models, by month, when analysis was censored in 2015. Mass drug administration/focal mass drug administration activities occurred in December 2014 and February 2015. May 2015 represents the end of the first rainy season period. This figure appears in color at www.ajtmh.org.

The mean time to first infection over the full 17-month follow-up period in the higher transmission areas was 14.0 months (95% CI: 13.4–14.5) in the MDA arm, 13.5 months (95% CI: 12.9–14.1) in the fMDA arm, and 12.9 months (95% CI: 12.2–13.6) in the control arm; in the low transmission areas, it was 16.6 (95% CI: 16.4–16.8), 16.5 (95% CI: 16.2–16.7), and 16.3 (95% CI: 16.0–16.6) in the MDA, fMDA, and control arms, respectively. Kaplan–Meier curves for PCR infection by arm and by transmission strata are shown in Figure 5 for the full study period as well as for the first rainy season. In the unadjusted Cox proportional hazards model over the first rainy season period, MDA was associated with a lower risk of infection compared with control (HR: 0.46; 95% CI: 0.19–1.14). In the adjusted model over the first rainy season period, this association was significant across strata combined (HR: 0.36; 95% CI: 0.16–0.80) (Table 5) and higher transmission strata (HR: 0.36; 95% CI: 0.16–0.82) but did not reach significance in the lower transmission strata. Baseline PCR positivity was strongly associated with greater risk of an infection during the first rainy season period (HR: 2.24; 95% CI: 1.49–3.37). There were no statistically significant differences by age or gender, but household IRS at baseline was associated with lower risk of infection. Lower elevation was also associated with higher risk of infection. Distance to water body was excluded because of collinearity with elevation, and distance to health facility was not associated with risk of infection. Differences between arms were greatest in the first several months following the second MDA round (February 2015) and decreased slowly thereafter (Figure 6). In the adjusted model over the full study period, there was no significant difference between arms for any strata (results not shown).

**Table 5:** Hazard ratios for time to first infection by PCR from adjusted Cox proportional hazards regression over first rainy season, for high and low strata and combined, Southern province, Zambia, December 2014–May 2015

	High transmission	Low transmission	Combined
Baseline PCR status			
PCR− (ref)			
PCR+	1.99 (1.31–3.03)*	8.06 (1.57–41.39)*	2.24 (1.49–3.37)†
Control (ref)			
Mass drug administration	0.36 (0.16–0.82)*	0.65 (0.16–2.66)	0.36 (0.16–0.80)*
Focal mass drug administration	0.49 (0.21–1.13)‡	1.22 (0.35–4.27)	0.52 (0.23–1.18)
Age category (years)			
< 5 (ref)			
5–20	1.17 (0.75–1.84)	0.62 (0.21–1.80)	1.07 (0.71–1.62)
> 20	0.99 (0.61–1.59)	0.76 (0.26–2.23)	0.96 (0.62–1.48)
Gender			
Male (ref)			
Female	0.78 (0.56–1.09)	1.09 (0.48–2.52)	0.83 (0.61–1.13)
Wealth quintile			
1 (ref)			
2	0.96 (0.59–1.58)	1.24 (0.30–5.11)	0.95 (0.60–1.51)
3	0.64 (0.39–1.07)	1.38 (0.37–5.06)	0.67 (0.42–1.08)†
4	0.70 (0.41–1.19)	0.50 (0.09–2.90)	0.63 (0.38–1.06)‡
5	0.66 (0.36–1.20)	1.11 (0.27–4.48)	0.67 (0.38–1.15)
IRS, first month	0.43 (0.18–1.04)‡	§	0.39 (0.17–0.94)*
Rainfall (SD)	1.24 (0.82–1.90)	8.18 (2.12–31.58)*	1.45 (0.99–2.12)‡
Environmental vegetation index (SD)	0.94 (0.70–1.25)	0.45 (0.19–1.05)‡	0.87 (0.66–1.14)
Altitude (SD)	0.54 (0.35–0.81)*	0.44 (0.25–0.75)*	0.41 (0.29–0.59)†

IRS = indoor residual spraying; PCR = polymerase chain reaction; SD = standardized to 1 SD.

* < 0.05.

† < 0.001.

‡ < 0.1.

§ Term removed because of zero new infections in households with IRS.

### Cumulative infection incidence models.

**Figure 7.**
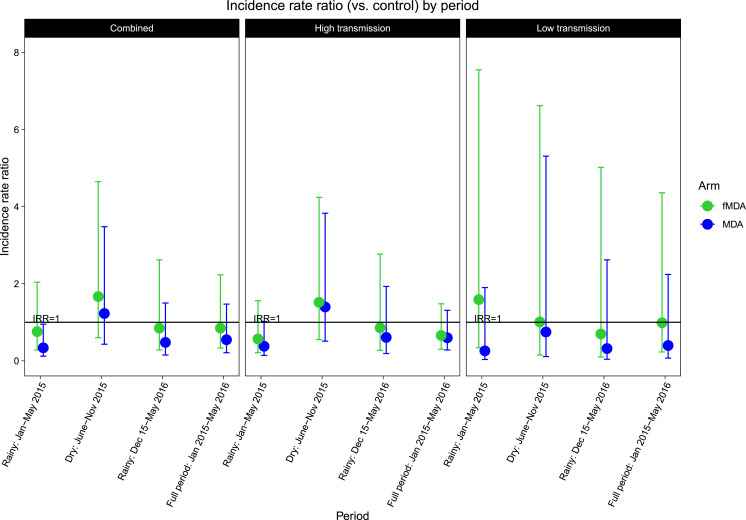
Incidence rate ratios (IRRs) for mass drug administration (MDA) and focal mass drug administration (fMDA) vs. control by time period (season) of follow-up based on adjusted negative binomial models. Mass drug administration/focal mass drug administration campaign rounds occurred in December 2014, February 2015, October 2015, and February 2016. From the adjusted model with a season by arm interaction term (Table 6), coefficients for MDA and fMDA were calculated for each season: the first rainy season (January–May 2015), the dry season (June–November 2015), and the second rainy season (December 2015–May 2016). The IRR for the full period of study is based on the corresponding adjusted model with no season by arm interaction term. This figure appears in color at www.ajtmh.org.

In the unadjusted model, MDA reduced cumulative infection incidence by PCR in the first rainy season but did not reach significance in combined strata (IRR: 0.30; 95% CI: 0.09–1.04) or in the higher transmission (IRR: 0.44; 95% CI: 0.15–1.35) or lower transmission strata by themselves (IRR: 0.21; 95% CI: 0.03–1.34). In the adjusted model, the effect of MDA reached significance in the combined strata in the first rainy season (IRR: 0.34; 95% CI: 0.12–0.95); this effect was slightly less pronounced in the high transmission stratum (IRR: 0.38; 95% CI: 0.14–1.02) and did not reach significance in the lower transmission stratum (IRR: 0.26; 95% CI: 0.04–1.90) (Table 6). Although the interaction terms between arm and time period were significant, neither MDA nor fMDA were significantly different from control in the dry season or second rainy season, or over the full study period (Figure 7). Baseline PCR status was strongly associated with follow-up infection incidence in the combined and high transmission strata. There were no significant differences by age or gender, but greater wealth, household IRS in the past 12 months at baseline, and higher elevation were associated with lower incidence.

**Table 6:** Results of adjusted negative binomial regression model of cumulative infection incidence by PCR with interaction term for time period of follow-up and study arm, for high and low strata and combined, Southern province, Zambia, December 2014–May 2016.

	High transmission	Low transmission	Combined
Baseline PCR status			
PCR−(ref)			
PCR+	1.60 (1.15–2.22)*	1.70 (0.24–11.98)	1.61 (1.15–2.26)*
Study arm			
Control (ref)			
MDA	0.38 (0.14–1.02)†	0.26 (0.04–1.90)	0.34 (0.12–0.95)‡
FMDA	0.57 (0.21–1.56)	1.59 (0.34–7.55)	0.76 (0.28–2.04)
Season			
Rainy (January–May 2015) (ref)			
Dry (June–November 2015)	1.13 (0.12–10.36)	0.00 (0.00–3.05)†	0.51 (0.06–4.23)
Rainy (December 15–May 2015)	0.28 (0.13–0.61)‡	0.11 (0.01–0.89)‡	0.27 (0.13–0.54)*
Season *×* study arm interaction			
Dry *×* MDA	3.69 (2.11–6.45)*	2.89 (0.45–18.50)	3.58 (2.12–6.04)*
Dry *×* fMDA	2.65 (1.50–4.71)‡	0.64 (0.12–3.26)	2.21 (1.30–3.77)‡
Rainy *×* MDA	1.61 (0.74–3.53)	1.25 (0.18–8.53)	1.40 (0.70–2.81)
Rainy *×* fMDA	1.51 (0.67–3.40)	0.44 (0.08–2.40)	1.13 (0.55–2.31)
Age category (years)			
< 5 (ref)			
5–20	1.15 (0.83–1.61)	1.05 (0.45–2.44)	1.17 (0.86–1.60)
> 20	1.12 (0.79–1.58)	0.82 (0.33–2.01)	1.10 (0.79–1.52)
Gender			
Male (ref)			
Female	0.89 (0.69–1.14)	1.17 (0.60–2.26)	0.92 (0.73–1.17)
Wealth quintile			
1 (ref)			
2	1.34 (0.92–1.95)	1.23 (0.40–3.85)	1.31 (0.91–1.88)
3	0.90 (0.62–1.29)	1.37 (0.50–3.79)	0.93 (0.66–1.32)
4	0.93 (0.62–1.39)	0.79 (0.24–2.58)	0.87 (0.59–1.28)
5	0.51 (0.31–0.83)‡	0.73 (0.23–2.32)	0.53 (0.34–0.83)‡
Indoor residual spraying, first month	0.60 (0.30–1.19)	0.30 (0.03–2.58)	0.54 (0.28–1.04)†
Rainfall (SD)	1.52 (0.63–3.71)	0.11 (0.00–3.44)	1.15 (0.49–2.68)
Environmental vegetation index (SD)	1.10 (0.86–1.41)	0.54 (0.25–1.17)	1.01 (0.80–1.27)
Altitude (SD)	0.42 (0.26–0.67)*	0.49 (0.26–0.92)‡	0.31 (0.20–0.47)*

fMDA = focal mass drug administration; PCR = polymerase chain reaction; MDA = mass drug administration; SD = standardized to 1 SD. Coefficients for MDA and fMDA for each season based on the interaction term are depicted in Figure 7.

* < 0.001.

† < 0.1

‡ < 0.05.

## DISCUSSION

In this analysis of longitudinal cohort data collected over an 18-month period, we observed that the MDA study arm was associated with an increased time to first infection and decreased cumulative infection incidence compared with the control arm over the first rainy season; these differences were not seen in the comparison of the fMDA arm versus the control. In addition, the effect of MDA was greatest in the first several months following the initial two campaign rounds and decreased over subsequent months. Of note, although we found greater evidence of a statistically significant effect in the higher transmission stratum, effect estimates were generally larger, albeit not statistically significant, in the lower transmission stratum because of the low number of infections observed. Simultaneous decreases in infection incidence in all study areas reduced study power for discerning significant intervention effects, particularly in lower transmission areas and over the entire 18-month period of study. Similar results were found for parasite prevalence,^8^ which fell from 31.3% to 4.0% between 2014 and 2016 across the study area, irrespective of the exposure group.

In the one previous community-randomized controlled trial of MDA conducted in the Gambia that included a longitudinal cohort, a 5-month follow-up period in children was conducted after a single round of MDA with sulfadoxine–pyrimethamine and artesunate. In that trial, MDA reduced incidence only for the first 6 weeks following treatment.^3,4^ Similarly, we found evidence that the effect of MDA or fMDA was greatest in the first 3 months following the second campaign round in February 2015, and diminished after that point.

The reduced ability to detect a statistically significant effect of either MDA or fMDA on time to first infection or cumulative infection incidence was influenced both by dramatic reductions in transmission across the study area as a whole and very low rates of infection during the traditional lower transmission season of June through November. The low number of infections, especially in the low transmission areas, during this season limited statistical assessment of effect. Similarly, the dramatic reduction in transmission in all study arms over the first two rounds of MDA limited our ability to detect an effect during the second two mass treatment rounds in the second year of the study.

Risk of infection was strongly associated with infection status at baseline at both the household and individual level, as has been found elsewhere,^12^ and suggests a relative stability of infection risk over time. Individuals who were parasitemic at baseline or residing in homes with a parasitemic individual regardless of their own status were significantly more likely to have an infection during follow-up than individuals who were non-parasitemic at baseline (or lived in households where nobody was parasitemic). In addition, these effects were far more pronounced in the low transmission stratum, which reflects the increasing clustering of infections in high-risk households as transmission decreases. Infection risk was not highly associated with age or gender but rather with lower household wealth status, lack of recent IRS, and environmental attributes including higher soil moisture (measured through vegetation indices) and lower altitude. As transmission decreases to low levels, improving the targeting of MDA and other interventions to specific households and areas that remain at higher risk of malaria is critical and may improve the cost-effectiveness and acceptability of these interventions.

The effect of MDA appeared more pronounced in individuals who were infected at baseline, suggesting that the bulk of the effect was due to clearing the baseline infection in these individuals as well as the prophylactic effect of DHAp in individuals in high-risk homes or areas. However, the effect of fMDA was less pronounced than for MDA across outcomes and transmission strata, likely due to the low diagnostic sensitivity of the RDT used to screen households; RDT sensitivity was found to be only 53% across all samples compared with PCR.^13^ A focal approach may prove more feasible and cost-effective in areas of low transmission, where household clustering is usually greater, and may be enhanced with the use of new, more sensitive diagnostics.^14–16^

This analysis was somewhat limited because of challenges related to long-term data collection at the community level by community health workers. Compared with several similar previous longitudinal studies,^17–20^ ours was substantially larger in terms of the numbers of individuals enrolled yet achieved higher rates of follow-up than similar studies of community-level testing and treatment in Zambia.^21^ That said, although a high proportion of individuals enrolled at baseline were followed up for at least a year, the proportion of individuals with complete follow-up data dropped substantially between 12 and 17 months. For monitoring trial outcomes, rotating cohort enrollment every 6–12 months should be considered to limit respondent fatigue and movement out of the study households. In addition, some variables, including fever, treatment, and travel status, were only available for a subset of individuals, limiting their inclusion in the risk factor analysis.

We used actively detected infections as our primary outcome, which is an improvement on passive methods that may be subject to reporting and treatment-seeking bias. However, we could not definitively determine whether all positive tests represented new infections, previous untreated infections, or residual parasite DNA or HRP2 from a recently treated infection. By removing all PCR-positive results that followed a PCR positive in the previous month where an RDT was not captured and treatment not given, we limited the possibility for this bias in the cumulative incidence analysis. In addition, clearance of parasites following treatment with DHAp was found to be 100% among 37 baseline participants, and genotyping on a subsample found several new infections that occurred just 2 months following an initial infection.^22^ Finally, the reduced power due to broad transmission decline across Southern Province was unexpected. In addition to transmission declines due to drier climate and scale-up of vector control, the reduction in infections in cohort households in the control arm may have been enhanced by greater coverage, especially at the community level, of routine testing and treatment with AL. Increased treatment-seeking behavior in cohort individuals also may have played a role.

In sum, we demonstrated short-term effects of MDA with DHAp in a large community-level cohort in Southern Province, Zambia. This study suggests MDA may serve as an accelerator of transmission reduction in areas that have already achieved high levels of vector control and access to case management, and that once burden is reduced through initial campaign rounds, a transition to targeting only the highest risk individuals and areas may be warranted to achieve subsequent reductions.

## References

[1] Miller JM Eisele TP Fraser MS Lewis MT Slutsker L Chizema Kawesha E, 2020 Moving from malaria burden reduction toward elimination: an evaluation of mass drug administration in Southern Province, Zambia. Am J Trop Med Hyg 103 (Suppl 2): 3–6. 10.4269/ajtmh.19-0669 PMC7416971 32618265

[2] Eisele TP 2015 Assessing the effectiveness of household-level focal mass drug administration and community-wide mass drug administration for reducing malaria parasite infection prevalence and incidence in Southern Province, Zambia: study protocol for a community randomized controlled trial. Trials 16: 347. 26268804 10.1186/s13063-015-0862-3 PMC4535296

[3] Von Seidlein L 2003 The effect of mass administration of sulfadoxine-pyrimethamine combined with artesunate on malaria incidence: a double-blind, community-randomized, placebo-controlled trial in the Gambia. Trans R Soc Trop Med Hyg 97: 217–225. 14584381 10.1016/s0035-9203(03)90125-8

[4] Poirot E Skarbinski J Sinclair D Kachur SP Slutsker L Hwang J, 2013 Mass drug administration for malaria. Cochrane Database Syst Rev 12: CD008846. 10.1002/14651858.CD008846.pub2 PMC4468927 24318836

[5] Mwesigwa L 2019 Mass drug administration with dihydroartemisinin-piperaquine and malaria transmission dynamics in the Gambia: a prospective cohort study. Clin Infect Dis 69: 278–286. 30304511 10.1093/cid/ciy870 PMC6603267

[6] Yukich J Briet O Bretscher MT Bennet A Lemma S Berhane Y Eisele TP Keating J Smith T, 2012 Estimating *Plasmodium falciparum* transmission rates in low-endemic settings using a combination of community prevalence and health facility data. PLoS One 7: e42861. 22936995 10.1371/journal.pone.0042861 PMC3425560

[7] Hay SI Smith DL Snow RW, 2008 Measuring malaria endemicity from intense to interrupted transmission. Lancet Infect Dis 8: 369–378. 18387849 10.1016/S1473-3099(08)70069-0 PMC2653619

[8] Eisele TP 2020 Impact of four rounds of mass drug administration with dihydroartemisinin-piperaquine implemented in Southern Province, Zambia. Am J Trop Med Hyg 103 (Suppl 2): 7–18. 10.4269/ajtmh.19-0659 PMC7416977 32618247

[9] WHO, 2017 Mass Drug Administration for Falciparum Malaria: a Practical Field Manual*.* Geneva, Switzerland: World Health Organization.

[10] Eisele TP 2016 Short-term impact of mass drug administration with dihydroartemisinin plus piperaquine on malaria in Southern Province Zambia: a cluster-randomized controlled trial. J Infect Dis 214: 1831–1839. 27923947 10.1093/infdis/jiw416 PMC5142084

[11] Funk C. 2015 The climate hazards infrared precipitation with stations—a new environmental record for monitoring extremes. Sci Data 2: 150066. 26646728 10.1038/sdata.2015.66 PMC4672685

[12] Cook J Owaga C Marube E Baidjoe A Stresman G Migiro R Cox J Drakeley C Stevenson JC, 2018 Risk factors for *Plasmodium falciparum* infection in the Kenyan Highlands: a cohort study. Trans R Soc Trop Med Hyg 1–8. 30496556 10.1093/trstmh/try122 PMC6391934

[13] Chishimba S 2020 Prevalence of *Plasmodium falciparum* and *non-falciparum* infections by photo-induced electron transfer-PCR in a longitudinal cohort of individuals enrolled in a mass drug administration trial in Southern Province, Zambia. Am J Trop Med Hyg 103 (Suppl 2): 82–89. 32618252 10.4269/ajtmh.19-0668 PMC7416980

[14] Das S 2017 Performance of a high-sensitivity rapid diagnostic test for *Plasmodium falciparum* malaria in asymptomatic individuals from Uganda and Myanmar and naive human challenge infections. Am J Trop Med Hyg 97: 1540–1550. 28820709 10.4269/ajtmh.17-0245 PMC5817764

[15] Yukich J 2017 Estimation of malaria parasite reservoir coverage using reactive case detection and active community fever screening from census data with rapid diagnostic tests in southern Zambia: a re-sampling approach. Malar J 16: 317. 28784122 10.1186/s12936-017-1962-1 PMC5547485

[16] Yukich JO 2020 Cost-effectiveness of focal mass drug administration and mass drug administration with dihydroartemisinin-piperaquine for malaria prevention in Southern Province, Zambia: results of a community-randomized controlled trial. Am J Trop Med Hyg 103 (Suppl 2): 46–53. 32618249 10.4269/ajtmh.19-0661 PMC7416981

[17] Wanzira H Kakuru A Arinaitwe E Bigira V Muhindo MK Conrad M Rosenthal PJ Kamya MR Tappero JW Dorsey G, 2014 Longitudinal outcomes in a cohort of Ugandan children randomized to artemether-lumefantrine versus dihydroartemisinin-piperaquine for the treatment of malaria. Clin Infect Dis 59: 509–516. 24825870 10.1093/cid/ciu353

[18] Trape JF 2014 The rise and fall of malaria in a West African rural community, Dielmo, Senegal, from 1990 to 2012: a 22 year longitudinal study. Lancet Infect Dis 14: 476–488. 24813159 10.1016/S1473-3099(14)70712-1

[19] Degefa T Zeynudin A Godesso A Michael YH Eba K Zemene E Emana D Birlie B Tushune K Yewhalaw D, 2015 Malaria incidence and assessment of entomological indices among resettled communities in Ethiopia: a longitudinal study. Malar J 14: 24. 25626598 10.1186/s12936-014-0532-z PMC4318213

[20] Simmons RA Mboera L Miranda ML Morris A Stresman G Turner EL Kramer R Drakeley C O’Meara WP, 2017 A longitudinal cohort study of malaria exposure and changing serostatus in a malaria endemic area of rural Tanzania. Malar J 16: 309. 28764717 10.1186/s12936-017-1945-2 PMC5539976

[21] Hamainza B Moonga H Sikaala CH Kamuliwo M Bennett A Eisele TP Miller J Seyoum A Killeen GF, 2014*.* Monitoring, characterization and control of chronic, symptomatic malaria infections in rural Zambia through monthly household visits by paid community health workers. Malar J 13: 128. 24678631 10.1186/1475-2875-13-128 PMC4113135

[22] Finn TP 2020 Adherence to mass drug administration with dihydroartemisinin-piperaquine and *Plasmodium falciparum* clearance in Southern Province, Zambia. Am J Trop Med Hyg 103 (Suppl 2): 37–45. 32618267 10.4269/ajtmh.19-0667 PMC7416972

